# The active knee extension after extensor mechanism reconstruction using allograft is not influenced by “early mobilization”: a systematic review and meta-analysis

**DOI:** 10.1186/s13018-022-03049-w

**Published:** 2022-03-09

**Authors:** Cristiano De Franco, Vincenzo de Matteo, Marco Lenzi, Ernesto Marano, Enrico Festa, Alessio Bernasconi, Francesco Smeraglia, Giovanni Balato

**Affiliations:** Department of Public Health, Orthopedic Unit, “Federico II” University, Via Sergio Pansini, 5 80130, Naples, Italy

**Keywords:** Extensor mechanism, Allograft, Mobilization, Rehabilitation, TKA

## Abstract

**Background:**

Postoperative rehabilitation after extensor mechanism reconstruction (EMR) with allograft following total knee arthroplasty (TKA) is not standardized. This meta-analysis aimed to evaluate the effectiveness of early and late knee mobilization after EMR. The range of motion (ROM) and extensor lag in both groups were also assessed as the secondary endpoint.

**Methods:**

Following the Preferred Reporting Items for Systematic Review and Meta-Analyses (PRISMA) guidelines, a systematic review of the literature was performed, including studies dealing with the use of allograft for EMR following TKA. Failure was defined as the persistence of extensor lag > 20°. Coleman Methodology Score and Methodological Index for Non-Randomized Studies (MINORS) score were used to assess the quality of studies included. The failure rate was set as the primary outcome in early (4 weeks) and late (8 weeks) mobilization groups after EMR with allograft. Secondary outcomes were postoperative extensor lag and ROM.

**Results:**

Twelve articles (129 knees) were finally selected for this meta-analysis. Late and early knee mobilization was described in five and seven studies, respectively. No difference was noted between both groups' failure rates (11/84 vs. 4/38, respectively; *p* = 0.69). The mean extensor lag at last follow-up was 9.1° ± 8.6 in the early mobilization group, and 6.5° ± 6.1 in the late mobilization group is not significantly different (p > 0.05). The mean postoperative knee flexion was 107.6° ± 6.5 and 104.8° ± 7 in the early and late mobilization group, respectively.

**Conclusion:**

While immobilization after EMR in TKA is mandatory to allow tissue healing, early knee mobilization after four weeks can be recommended with no additional risk of failure and increased extensor lag compared to a late mobilization protocol.

**Level of evidence:**

IV, therapeutic study.

*Registration*

PROSPERO (International Prospective Register of Systematic Reviews): CRD42019141574.

## - Introduction

Extensor mechanism (EM) rupture during total knee arthroplasty (TKA) is a serious complication occurring in 0.1–2.5% procedures [1–3]. It may lead to a loss of active knee extension with an inability to perform straight-leg raise, which is often followed by a secondary reduction in the range of motion (ROM) complicated by knee instability, chronic pain, and recurrent falls. In turn, the limited knee function may significantly impact and reduce the overall quality of life [4, 5]. Although several surgical techniques have been proposed to approach EM rupture, there is no consensus on the gold standard for EM reconstruction (EMR) [6–9]. Therefore, the choice of surgical treatment depends on several factors, including the nature and chronicity of disruption, location of the failure, presence or absence of functional loss, general health status, and surgeon expertise [10]. According to the literature, the use of allografts to fill large defects during EMR [7–9, 11] has been proven to be effective, mainly due to the inherent mechanical strength of allografts and possibility of representing a fibrous scaffold which can subsequently be colonised by the host tissue. In fact, retrieval studies after EMR have shown incorporation of the host tissue into the allograft, providing insights into the effectiveness of this technique from a biological standpoint [12–15].

In clinical practice, a 4-week immobilisation period in extension of the knee is mandatory after EMR to allow wound and tissue healing. Weight-bearing restrictions may vary from non-weight bearing to partial weight-bearing, depending on the type of reconstruction performed and the patients’ related factors. The principles introduced by Nazarian and Booth, who first standardised the technique, are universally adopted and have allowed to improve the outcomes compared to previous procedures [16]. Among them, we emphasise the immobilisation of the knee in extension using a cast for 6 weeks, followed by flexion exercises with a 30° increase every 2 weeks, use of an articulated splint for an additional 6 to 12 weeks, and no pulley therapy or work against resistance for the first 6–12 months after removal of the cast. However, it has been argued that immobilisation of the knee for 4 or 8 weeks could generate post-operative stiffness with adherence and arthrofibrosis [17, 18]. Therefore, other authors have proposed early rehabilitation after 4 weeks, but there is no evidence of superiority of a protocol over another.

In this context, we performed a meta-analysis to evaluate the effectiveness of early mobilisation after EMR with allografts. Knee postoperative flexion and extensor lag in the early and late mobilisation groups were also evaluated.

## - Materials and methods

### - Registration

The protocol was registered online at the International Prospective Register of Systematic Reviews (PROSPERO; CRD42019141574) [19] before commencing the review.

### Allografts

The EM was reconstructed using two primary forms of allografts—Achilles tendon with attached calcaneal bone block and complete EM allograft comprising the proximal tibia, the patellar tendon, the patella, and several centimetres of the quadriceps tendon.

### Searches

Electronic databases, such as MEDLINE, Scopus, Embase, Web of Science, and Cochrane, were searched for studies investigating EMR in patients who underwent primary or revision TKA. The Preferred Reporting Items for Systematic Review and Meta-Analyses (PRISMA) methodology [20] was employed. A combination of the following keywords was used for article search: ‘Extensor mechanism’ AND ‘Allograft’ And ‘total knee arthroplasty’. The inclusion criteria were not limited to English language literature and specific publication dates. The reference lists of selected articles were searched for additional articles that were not identified in the database search.

### Study inclusion and exclusion criteria

Longitudinal studies (retrospective and prospective) and randomised controlled trials evaluating patients treated with allografts and concomitant TKA or revision TKA for EM rupture were included. Case reports, expert opinions, prior systematic reviews, letters to the editor, studies that did not include patients undergoing TKA, studies that included different treatment techniques (such as other allografts, synthetic mesh, or autograft), studies in which the postoperative treatment protocol was not specified, and non-human studies such as in vitro studies and cadaveric studies were excluded.

### Study quality assessment

The methodological quality of the included studies was assessed using the Coleman Methodology Score (CMS) and the Methodological Index for Non-Randomized Studies (MINORS) score [21, 22]. Two authors independently determined the CMS and MINORS score. The final scores were obtained through consensus. The CMS was computed by summation of 10 criteria (study size, follow-up period, number of procedures, study type, diagnostic certainty, description of surgical technique, rehabilitation and compliance, outcome criteria, outcome assessment, and selection process), leading to a total possible score of 100. Thus, the CMS ranged from 0 to 100. A higher score was associated with a lower probability that outcomes were caused by chance, bias, or confounding factors. MINORS score is a valid tool designed to assess the methodological quality of non-randomised surgical studies. The maximum MINORS scores for non-comparative and comparative studies were 16 and 24, respectively.

### Data extraction strategy

Initially, the titles and abstracts of the studies were screened by two independent reviewers. Full text was obtained for articles whose abstracts meet the inclusion criteria or those without any uncertainty. Then, each study was assessed based on the inclusion criteria by two independent reviewers, and any disagreement regarding inclusion of a particular study was resolved by evaluation of the article by the senior author. Relevant data were extracted from each study. Data on participant demographics, sample size, type of allograft used, site of injury, type of rehabilitation protocol, failure outcomes, and clinical and functional outcomes were recorded.

### Rehabilitation protocol

The included patients were divided into two groups based on the rehabilitation protocol adopted. In the first group (early mobilisation), passive and active knee mobilisation was initiated at the end of week 4, followed by a weekly increase of 15°–30° until week 12. In the second group (late mobilisation), mobilisation of the knee was performed 8 weeks after surgical repair, with a weekly increase until week 12. In both groups, a cast or splint was used, and weight-bearing and isometric exercises of the quadriceps were allowed (partially or based on tolerance).

### Data synthesis and presentation

The ‘failure rate’ was defined as the percentage of patients presenting a deficit of active knee extension (extensor lag) > 20°. The failure rate, with a 95% confidence interval (CI), was the primary outcome of the rehabilitation protocol after EMR with an allograft. The secondary outcomes were the ROM and extensor lag recorded at the last follow-up. Heterogeneity between studies was tested using the *I*^2^ statistic (0–40% = not relevant, 30–60% = moderate, 50–90% = substantial, and 75–100% = considerable). The primary outcome was pooled using random-effects models to determine the effect of interstudy heterogeneity. The chi-square test was used to analyze the significant cross-sectional differences between the two groups for the primary outcome. The two-sample t-test was used to analyze significant differences between the two groups for secondary outcomes. We used Open Meta Analyst (Centre for Evidence Synthesis, RI, USA) and SPSS version 23 (SPSS, Chicago, IL, USA) for all statistical analyses. Statistical significance was set at *p* ≤ 0.05.

## Results

### Review statistics

**Fig. 1:**
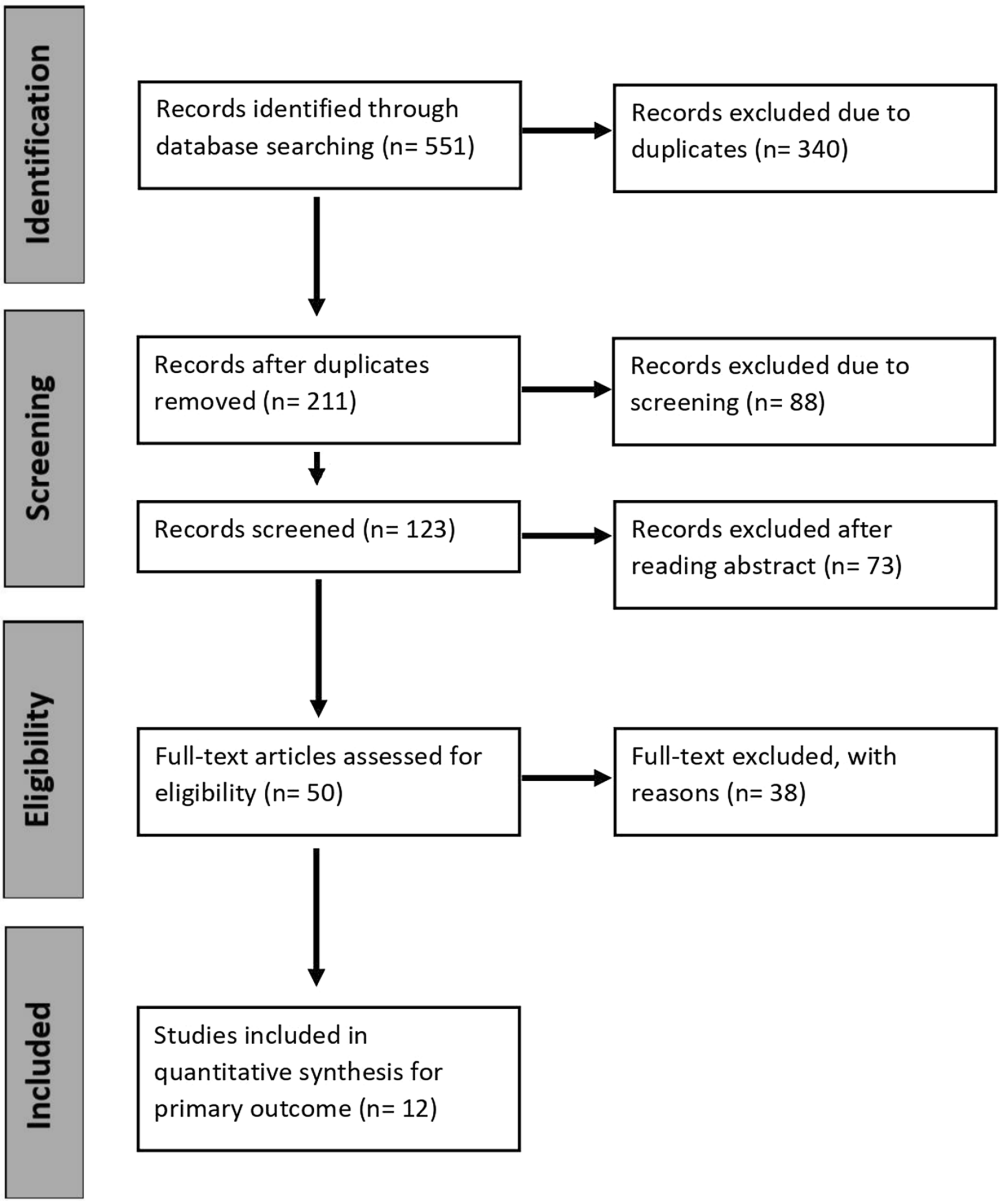
PRISMA flow chart diagram: search strategy

The PRISMA checklist is shown in Fig. 1. A total of 551 potentially relevant studies were identified through a computer search and manual screening of reference lists. After screening the titles and abstracts, the full texts of 50 articles were evaluated. A total of 38 studies were excluded after detailed assessment. The remaining 12 articles were included in the meta-analysis [23–34]. Table 1 summarises the characteristics of the included studies. A total of 126 patients (129 knees; weighted mean age, 68.1 ± 4 years) who underwent EMR with an allograft were identified. Among 120 patients, 83 (69%) were women. The weighted mean follow-up period was 3.4 ± 1.2 years.

**Table 1:** Basic Information of the Studies Included in the Systematic review

Author	Journal	No. of knees/patients	Type allograft	Site of injury	Age, mean	Female gender N (%)	Follow-up, mean, year	Coleman score	MINORS score
Boettner et al. [24]	Knee Surgery, Sports Traumatology, Arthroscopy	3/3	ATA*	3 Patella	67	2 (67%)	4	50	10
Burnett et al. [27]	Clinical Orthopaedics and Related Research	19/19	10 ATA*	8 Patellar tendon 2 Patella	66,1	7 (70%)	4.7	53	16
			9 EMA*	5 Patellar tendon 4 Patella	66,4	6 (67%)	4,5		
Wise et al. [33]	International Orthopaedics	17/16	ATA*	10 Patellar tendon 7 Quadriceps tendon	71	11 (69%)	3,8	53	12
Ares et al. [23]	Archives of Orthopaedic and Trauma Surgery	5/5	ATA*	5 Patellar tendon	74	4 (80%)	2,1	57	10
Crossett et al. [26]	The Journal of Bone and Joint Surgery	9/9	ATA*	9 Patellar tendon	70	6 (67%)	2	48	10
Lim et al. [30]	The Journal of Arthroplasty	16/16	12 ATA*	9 Patellar tendon 3 Quadriceps tendon	63	7 (58%)	2.9	43	10
			4 EMA*	4 Patellar tendon	71	2 (50%)	4.7		
Burnett et al. [25]	The Journal of Bone and Joint Surgery	13/13	EMA*	13 Patellar tendon 3 Quadriceps tendon 4 Patella	64	9 (69%)	3.1	50	17
Emerson et al. [28]	Clinical Orthopaedics and Related Research	15/14	9 EMA** 6 EMA*	15 Patellar tendon	69	12 (86%)	4.1	53	12
Leopold et al. [29]	The Journal of Bone and Joint Surgery	7/6	EMA*	7 Patellar tendon	73	N/A	3.3	55	13
Malhotra et al. [31]	The Journal of Arthroplasty	4/4	EMA* **	4 Patellar tendon	67,7	3 (75%)	1.8	50	13
Rajgopal et al. [32]	Journal of Knee Surgery	7/7	EMA*	7 Patellar tendon	60	5 (71%)	1,5	48	10
Wood et al. [34]	Journal of the American Academy of Orthopaedic Surgeons	14/14	4 EMA* 2 EMA**/6 ATA*	N/A	71	9 (64%)	5,3	63	12

ATA: Achilles tendon allograft; EMA: Entire extensor mechanism allograft; N/A: Not Available

^*^fresh frozen non-irradiated **freeze-dried

### Study quality assessment

The CMS and MINORS score for the included studies are shown in Table 1.

### Quantitative synthesis/meta-analysis

All studies included in our meta-analysis clearly described the rehabilitation protocol after the surgical procedure in terms of duration of knee immobilisation in full extension, type (active or passive) and timing of knee mobilisation, and weight-bearing status after the surgical procedure. They all evaluated functional outcomes (extensor lag and range of motion) at the last follow-up (Table 2).

**Table 2:** Knee mobilization and clinical outcomes

Author	Knee mobilization at 4 weeks	Knee mobilization at 8 weeks	Extensor lag at last FU	Knee flexion at last FU	Failures (lag > 20%)
Boettner et al. [24]	CPM 0–90	Free ROM	0 (0–0)	117 (110–125)	0 (0%)
Burnett et al. [27]	ROM 0–45	ROM 0–90	13.9 (0–90)	98 (80–100)	0 (0%)
Wise et al. [33]	ROM 0–45	ROM 0–90	6.6 (0–55)	105 (90–120)	4 (24%)
Ares et al. [23]	No	ROM 0–45	0 (0–0)	102 (95–110)	0 (0%)
Crossett et al. [26]	ROM 0–40	ROM 0–90	3 (0–20)	107 (95–125)	1 (11%)
Lim et al. [30] (12 ATA)	Isometric exercises	ROM 0–30	14.5 (0–60)	111 (0–130)	3 (25%)
Lim et al. [30] (4 EMA)	Isometric exercises	ROM 0–30	11.2 (0–40)	112 (90–130)	1 (25%)
Burnett et al. [25]	Isometric exercises	ROM 0–30	4.3 (0–15)	104 (85–122)	0 (0%)
Emerson et al. [28]	ROM 0–60	ROM 0–90	9.4 (0–40)	106 (80–130)	3 (27%)
Leopold et al. [29]	Isometric exercises	Gradual ROM	59 (40–80)	108 (90–130)	7 (100%)
Malhotra et al. [31]	Isometric exercises	Isometric exercises	2.5 (0–10)	95 (90–100)	0 (0%)
Rajgopal et al. [32]	ROM 0–15	ROM 0–45	5 (0–15)	115 (100–130)	0 (0%)
Wood et al. [34]	Gradual ROM	Gradual ROM	26 (± 15.5)	105 (± 20)	3 (23%)

CPM: continuous passive motion; FU: follow-up; ROM: range of motion

**Fig. 2:**
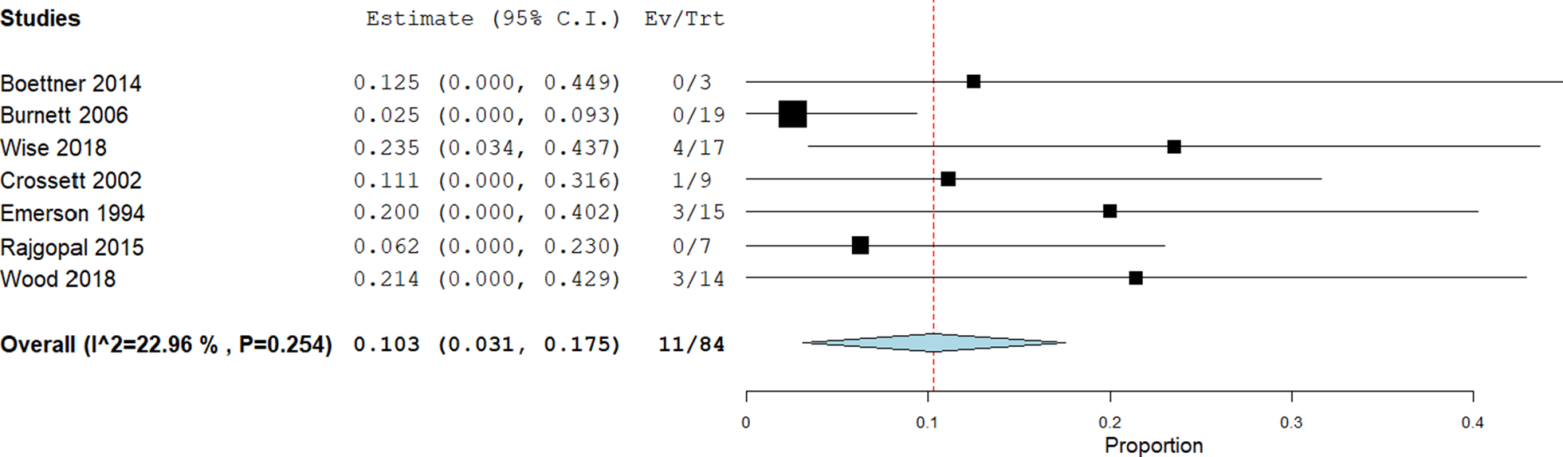
Early mobilization failure rate: forest plot describing the failure rate in case of early mobilization

Seven studies (84 knees) reported the failure rate of EMR after early mobilisation (4 weeks) in 11 cases, with a pooled failure rate of 10.3% (95% CI 3.1–17.5) and a non-significant interstudy heterogeneity (*I*^2^ = 22.96%; *p* = 0.254; Fig. 2). No difference was observed in the failure rate between early and late mobilisation (11/84 vs. 4/38; *p* = 0.69).

**Fig. 3:**
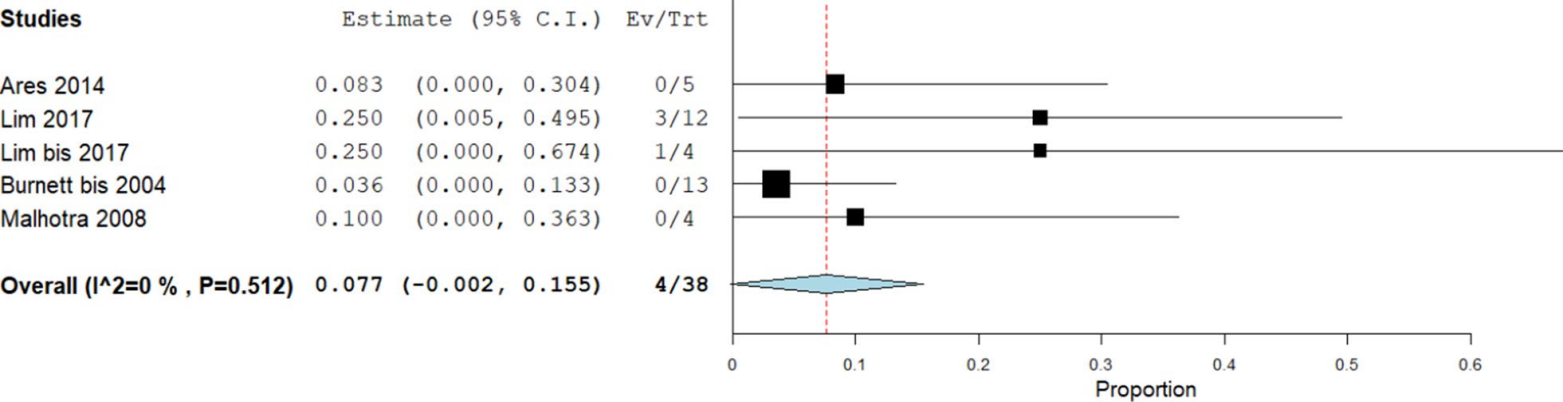
Late mobilization failure rate: forest plot describing the failure rate in case of late mobilization

Five studies (45 knees) reported the failure rate of EMR after late mobilisation (8 weeks). Failure of EMR was reported in 11 cases, and the corresponding pooled failure rate was 28% (95% CI 5.9–61.6), with a high heterogeneity between the included studies (*I*^2^ = 94.2%; *p* < 0.001). After exclusion of the outlier study by Leopold et al., the pooled failure rate decreased to 7.7% (95% CI 0.2–15.5), and the interstudy heterogeneity became nonsignificant (*I*^2^ = 0%; *p* = 0.512; Fig. 3).

The weighted mean extensor lag at the last follow-up was 6.5° ± 6.1° and 9.1° ± 8.6° for late and early mobilisation, respectively. No differences in extensor lag were noted between the two groups (*p* = 0.575).

Based on the available data, the postoperative weighted mean knee flexion was not significantly different between the two groups, but early mobilisation was associated with higher knee flexion at the longest follow-up than late mobilisation (107.6° ± 6.5° vs. 104.8° ± 7°; *p* = 0.495).

With regard to complications, in the early mobilisation group, we found four re-ruptures and five infections, while in the late mobilisation group, we found four re-ruptures and two infections, with no statistically significant difference between the two groups (*p* = 0.778 for re-ruptures and *p* = 0.515 for infections).

## - Discussion

Although EMR using allografts has the potential to improve EM function, augment host tissue, maintain ROM, and decrease dependence on walking aids, the postoperative rehabilitation protocol has not been standardised.

Immobilisation of the knee in full extension for a period of 4–8 weeks has been recommended for several reasons. First, the use of allografts requires immobilisation of the knee after surgery to promote healing of superficial and deep tissue and osteointegration of the graft in a weakened host tissue [12, 35]. Second, wound healing is an important parameter to be considered after this type of surgery, and early mobilisation of the knee during the initial post-operative days could stress superficial and deep tissues and consequently induce wound complications, especially in patients undergoing revision surgery [36–38].

However, prolonged post-operative immobilisation could also result in excessive scarring, with arthrofibrosis and joint stiffness [17]. Therefore, many surgeons have described an ‘early’ post-operative knee mobilisation after a 4-week period, promoting stretching of the muscle fibres with elongation of sarcomeres and a higher chance of recovering greater muscular strength at follow-up [39]. Nevertheless, early mobilisation could also stretch the tendon fibres of allografts, with a risk of elongation of the tendon and an incomplete return to its original length, especially in elderly patients [40].

This meta-analysis showed that early knee mobilisation was not associated with an increased risk of an extensor lag > 20°, with a failure rate of 10.3% and 7.7% in the early and late mobilisation groups, respectively. Therefore, our results suggest that early mobilisation does not negatively impact active knee extension at follow-up. Notably, in this study, we arbitrarily decided to exclude the study by Leopold et al. from the early mobilisation group; it described treatment failure in all six patients treated with EMA allografts [29]. The authors obtained mediocre outcomes with marked residual deficiencies in active knee extension mainly due to inadequate allograft tension, which allowed a flexion of approximately 60°.

The results of this study are in line with those presented by Boettner et al., who described the beginning of passive knee flexion through the application of continuous passive motion the day after the surgery or as soon as the soft tissue allowed [24]. The authors did not report any failures at follow-up.

Based on available data, the post-operative rehabilitation regimen does not influence extensor lag and ROM at follow-up, although early mobilisation has been reported to be associated with a greater knee flexion range at follow-up; therefore, late knee mobilisation after EMR with an allograft seems unnecessary.

The strengths and potential limitations of this study must be acknowledged. To the best of our knowledge, this is the first meta-analysis to evaluate the effectiveness of early mobilisation after EMR with an allograft in terms of failure rate, post-operative extensor lag, and knee flexion. First, this meta-analysis was performed on level II or level IV small case series. Second, the lack of standardisation between studies regarding the site of rupture, surgical technique, type of fixation, and postoperative protocols may have contributed to the heterogeneity of results between studies. This limitation prevented us from drawing a solid conclusion regarding the best postoperative rehabilitation protocol.

## - Conclusion

In conclusion, immobilisation after EMR is mandatory to allow tissue healing, but knee mobilisation after 4 weeks can be performed without a higher risk of failure and increased extensor lag compared to mobilisation after 8 weeks. However, early mobilisation is associated with a greater knee flexion range at follow-up.

## Data Availability

Not applicable.

## Abbreviations

ROM: Range of motion
TKA: Total knee arthroplasty
EM: Extensor mechanism
ATA: Achilles tendon allograft
EMA: Extensor mechanism allograft
CMS: Coleman Methodology Score
MINORS: Methodological Index for Non-randomized Studies
CPM: Continuous passive motion
